# Clinical Significance of Preoperative Assessment of Intravesical Prostatic Protrusion in Radical Prostatectomy

**DOI:** 10.7150/jca.86582

**Published:** 2023-09-11

**Authors:** Jie Tang, Wei Xi, Yanjun Zhu, Hang Wang, Li'an Sun, Shuai Jiang, Jianming Guo

**Affiliations:** 1 Department of Anesthesiology, Zhongshan Hospital, Fudan University, Shanghai 200032, China.; 2 Department of Urology, Zhongshan Hospital, Fudan University, Shanghai 200032, China.; 3 Department of Urology, Zhongshan Hospital Wusong Branch, Fudan University, Shanghai 200940, China.; Jie Tang, Wei Xi, and Yanjun Zhu contributed equally to the work.

**Keywords:** prostate cancer, intravesical prostatic protrusion, prostate-bladder junction, clinical significance, surgical margin of bladder neck.

## Abstract

**Background:** Intravesical prostatic protrusion (IPP) is common in prostate-related diseases, whose clinical significance in radical prostatectomy was unknown.

**Methods:** 791 patients underwent robot-assisted or open radical prostatectomy at our institution were enrolled. The transabdominal ultrasound examination of prostate and IPP was carried out preoperatively, by which IPP was classified as no (0-0.5cm, grade 0), slight (0.6-1.0cm, grade 1) and noticeable (>1.0cm, grade 2).

**Results:** 185 (23.4%), 170 (21.5%) and 436 (55.1%) patients had no, slight and noticeable IPP, respectively. Generally, prostate specific antigen (PSA), Gleason score and pT stage increased with IPP grade. In particular, cases with grade 0 IPP had a decreased proportion of seminal vesicles' involvement than those with grade 1 and grade 2 IPP (p=0.035). Reconstruction of the bladder neck (in robot-assisted group), increased surgical bleeding (>200ml), and prolonged postoperative hospital stays (>14 days) happened more in patients with grade 2 IPP. Blood transfusion only happened in patients with noticeable IPP. PSM of bladder neck was only associated with higher IPP grade in open surgery group (p=0.032), not in robot-assisted surgery group.

**Conclusion:** IPP is associated with cancer aggressiveness, surgery difficulty and PSM of bladder neck in prostate cancer. Assessment of it provides more information for operations.

## Introduction

Prostate cancer is one of the most common malignancies in men. Over 174,000 cases are estimated to be diagnosed of it annually, accounting for 20% of all new malignancies in male adults^1^. Radical prostatectomy is an optimal choice in treating resectable non-metastatic disease. Anatomic examinations of the prostate before surgery are useful because they not only provide information upon surgery but also give hints on cancer aggressiveness.

Intravesical prostatic protrusion (IPP) is an anatomic phenomenon of prostate. In benign prostatic hyperplasia (BPH), it reflects bladder outlet obstruction, hints medication response, gives rise to hydronephrosis and is useful in perioperative assessment^2^-^4^. In prostate cancer, its association with early recovery of continence after prostatectomy was consistently clarified 5, 6. Xu et al.^7^ found that combining IPP with prostate specific antigen (PSA) was useful in distinguishing malignancies from the crowd. However, related research of IPP in prostate cancer is limited and some fundamental issues remain unknown. First, the close relation between IPP and the bladder neck may have an impact on surgery. Then is IPP associated with indicators reflecting surgery difficulty, such as reconstruction of the bladder neck, bleeding, blood transfusion and postoperative hospital stay? Second, is IPP associated with cancer aggressiveness, such as TNM stage, Gleason score and PSA level. Third, IPP is strongly associated with PV, do they have similar clinical significance? We sought to investigate into these issues in the present study.

## Materials and Methods

A total of 940 patients who underwent robot-assisted or open radical prostatectomy from a single medical team in our hospital between 2013 and 2017 were reviewed. All patients received assessments of total PSA, emission computed tomography and transabdominal ultrasound examination of the prostate. Prostate volume and IPP were described with ultrasound examination. Patients with bone metastasis, rare pathology type, inadequate clinicopathological information and unqualified ultrasound reports were excluded. Finally, a total of 791 patients were enrolled into the study.

Clinicopathological information was collected from archived records. Gleason score, involvement of both lobes and seminal vesicle(s), microvascular invasion, prostate capsule invasion, extracapsular extension, lymph node involvement and PSM were faithfully recorded from pathology reports. T stage was redetermined by 2002 AJCC classification. Reconstruction of the bladder neck and operational bleeding were determined according to surgery records. Reconstruction of the bladder neck refers to narrowing the neck with suturing to make it fit to urethral edge. With transabdominal ultrasound examination, IPP was measured and classified as no (0-0.5cm, grade 0), slight (0.6-1.0cm, grade 1) and noticeable (>1.0cm, grade 2).

For statistical analysis, PV was classified into three groups given the experience in the literature and the distribution in our study. We used the Kruskal-Wallis test, Pearson's χ2 test and Fisher's exact test to evaluate associations among listed variables. P<0.05 was considered to be significant. All analysis was done with IBM SPSS Statistics 21, and figures were conducted with GraphPad Prism 6. Clinical Research Ethics Committee of our hospital authorized this study.

## Results

### Baseline characteristics

Baseline characteristics were shown in Table 1. Median age was 68 years (IQR: 64-72). 80.3% and 19.7% patients were performed with robot-assisted and open surgery, respectively. With transabdominal ultrasound examination, 185 (23.4%), 170 (21.5%) and 436 (55.1%) patients had no, slight and noticeable IPP, respectively. It was strongly associated with PV as expected (21.2ml, 25.5ml and 40.9ml, p<0.001).

### With cancer aggressiveness

Generally, higher IPP grade was associated with higher cancer grade, stage and preoperative tPSA. Gleason ≥8 was 20.5%, 22.9% and 33.3% (p=0.001), pT ≥2c was 68.1%, 71.2% and 78.2% (p=0.018) in those with 0, 1 and 2 IPP, respectively. Further assessment of variables affecting pT stage according to AJCC classification revealed that increased proportion of involvement of both lobes (64.3% in grade 0, 65.8% in grade 1 and 75.0% in grade 2, p=0.009) and seminal vesicles (16.7% in grade 0, 27.6% in grade 1 and 24.7% in grade 2, p=0.035) were responsible for the association between IPP and pT stage (Table 1). PSA also increased with IPP grade that median PSA was 12.0, 15.2 and 16.0 ng/ml in grade 0, 1 and 2 IPP patients (p=0.025). 1.6%, 5.9% and 3.7% of patients with no, slight and noticeable IPP had lymph node involvement with no statistical significance (p=0.093). Therefore, IPP was positively associated with cancer stage, pathological grade as well as PSA level, indicating that higher IPP hinted increased aggressiveness of prostate cancer.

### With surgery difficulty related indicators

Since IPP could affect the detachment of the bladder neck, we studied the association with indicators reflecting surgery difficulty, including reconstruction proportion of the bladder neck, bleeding, blood transfusion and postoperative hospital stay.

**Figure 1:**
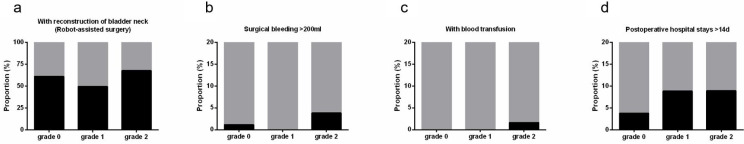
Association between IPP and surgical indicators. (a) with reconstruction of bladder neck in patients with robot-associated surgery; (b) with surgical bleeding >200ml; (c) with blood transfusion; (d) with postoperative hospital stays>14d.

Nearly all patients (91%) who underwent open surgery received reconstruction of the bladder neck, whereas the proportion was 62.2% in robot-assisted surgery group. The proportion of patients with grade 0, 1 and 2 IPP was 61.0%, 49.6% and 67.6%, respectively (Figure 1a).

Across all the patients, 18 (2.4%) had surgical bleeding over 200ml. Its distribution among IPP groups was distinct that patients with grade 2 IPP had the highest proportion (grade 2, 1 and 0: 3.8%, 0 and 1.1%, p=0.002, Figure 1b). In open surgery, the proportion was 6.9%, 0 and 2.1%, respectively (p=0.126). In robot-assisted surgery, the proportion was 3.2%, 0 and 0.8%, respectively (p=0.011). Seven out of 791 patients received blood transfusion during surgery. They all had grade 2 IPP (Figure 1c).

The median postoperative hospital stay was 6.0 days. 61 patients (7.7%) had prolonged postoperative hospital stays (≥14d), which happened more in patients with grade 2 or 1 IPP (>14d: 8.9% vs 3.8%, p=0.022, Figure 1d).

Therefore, higher IPP grade was associated with increased chance of reconstruction of the bladder neck (in robot-assisted surgery), increased bleeding (>200ml), blood transfusion as well as prolonged postoperative hospital time.

### With PSM of bladder neck

**Figure 2:**
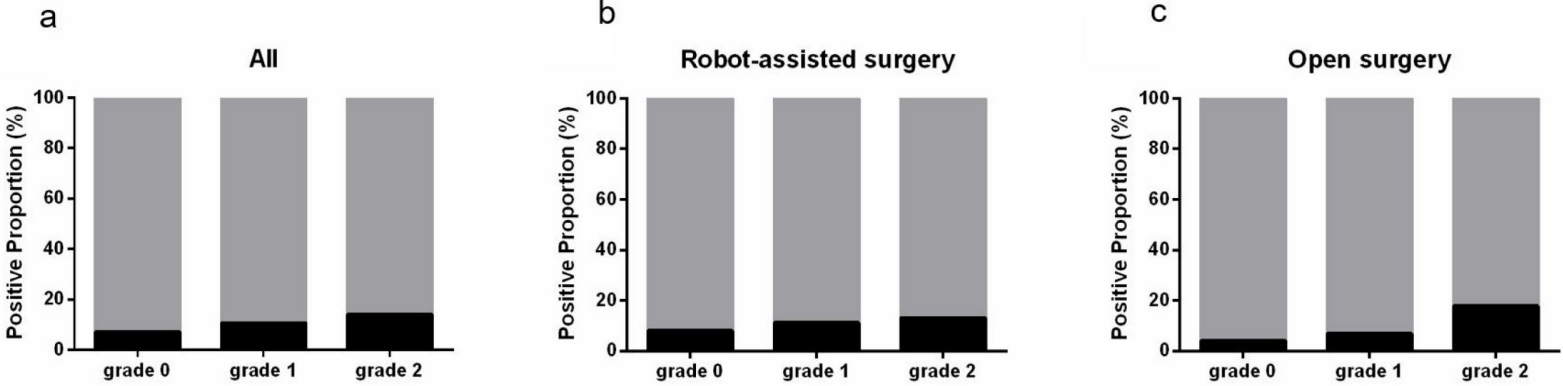
Association between IPP and PSM of bladder neck. (a) in all patients; (b) in robot-assisted surgery group; (c) in open surgery group.

The proportion of PSM of bladder neck increased with IPP grade across all the patients (7.0%, 10.6% and 14.0% in grade 0, 1 and 2 IPP, p=0.042). The association was more obvious in open surgery group (4.1%, 6.9% and 17.9%, p=0.032) but insignificant in robot-assisted surgery group (8.1%, 11.3% and 13.1%, p=0.294) (Figure 2).

## Discussion

The present study focused on the significance of preoperative assessment of IPP in men underwent radical prostatectomy. To achieve it, we studied the associations of IPP with cancer aggressiveness, surgery related indicators and PSM of the bladder neck. First, IPP was positively associated cancer aggressiveness. Increasing in IPP grade indicated more probability of high grade and stage. Second, IPP indicated more need for reconstruction of bladder and more bleeding in surgery. Postoperative hospital stays after surgery was also prolonged. In open surgery, high IPP grade was also associated with PSM of bladder neck. As far as we know, it is the first report focusing on the clinical significance of IPP in radical prostatectomy.

Volume of prostate was consistently reported to be associated with aggressiveness of prostate cancer. In a large cohort with 5657 cases, Moschini et al.^8^ reported that patients with PV>63ml had lower Gleason score, pT stage, extracapsular extension and BCR. Similar association of PV with cancer grade and stage was also detected in other reports, but its implications for oncological outcomes were obscure^9^-^11^. Although IPP was little studied in prostate cancer. Its strong correlation with PV gave us an instinct that IPP was also associated with lower grade and stage, which was disproved by our study. In the present study, we found that although higher IPP grade and larger PV were associated with increased PSA level, they had distinct meaning for cancer aggressiveness. Although PV was not associated with pT stage in our study, it was strongly associated with Gleason score and involvement of micro-nerve fibers (Table S1). Similar contradiction between IPP and PV was also reported in distinguishing prostate cancer from populations in the literature, which is an indirect validation of our findings. Shadi et al.^12^ reviewed 448 patients who had biopsy and found that those with a small prostate had much higher positive biopsy rate than those with a larger one (66% vs 40%, p<0.001). In another cohort of Chinese population (n=1486), the positive rates of biopsy in PV<35ml, 35-50ml, 50-67ml, 67ml were 48.2%, 28.1%, 13.7% and 8.4%, respectively^13^. So, PV was negatively associated with incidence of prostate cancer. On the contrary, IPP was positively associated with it. In patients with PSA of “grey zone” (4.0-10.0 ng/ml, n=339), IPP was indicated to be associated with positive rate of prostate cancer and integrating it with tPSA could significantly increase the predictive accuracy^7^. Therefore, the strong relation between PV and IPP did not guarantee their similar indications for cancer aggressiveness. Surgeons should pay more attention to involvement of seminal vesicles in cases with noticeable IPP during operation because their strong correlation. One possible explanation is that the seminal vesicle is located above the posterior prostate, and the presence of IPP may increase the potential contacting area between the vesicles and prostate.

IPP is a morphological and noticeable change at the junction with bladder. In surgery, dissection of the junction is often affected by IPP. High IPP grade could result in more bleeding and need for reconstruction of the bladder neck. The assumption was firstly verified by the present study. Proportion of increased bleeding was much higher in those with noticeable IPP than cases with slight IPP or without. The phenomenon both existed in open and robot-assisted groups albeit only 156 case with open surgery. All seven patients with blood transfusion had noticeable IPP, only two of them had a volume >50ml (82ml and 136ml). On the contrary, PV was not found to be associated with reconstruction of bladder neck (robot-assisted surgery), surgical bleeding >200ml, blood transfusion as well as postoperative hospital stays >14d (Table S2). Therefore, higher IPP grade increased surgery difficulty. Assessment of IPP could provide more information before surgery which cannot be replaced by PV.

PSM could increase the risk of adverse oncological outcome. In a recent meta-analysis involving 141,222 cases, PSM resulted in 35% more risk of BCR, 23% more risk of cancer-specific mortality and 9% more risk of overall mortality^14^. Approximately 20%-35% of all cases had PSM after surgery. Of them, the apex and the bladder neck were mostly involved^15^. Given the anatomic relationship, a spontaneous assumption is that IPP could result in change of PSM rate. It was verified in the study. In open surgery, PSM rate was 17.9% in patients with noticeable IPP whereas the proportion was only 6.9% in those with slight IPP and 4.1% in those without IPP. The finding was not obvious in robot-assisted group although a slight tendency was observed. We speculate that this phenomenon could be related to surgical convenience in robot-assisted surgery. Procedure was far optimized by the flexibility of robot arms, which makes the resection line close to the prostate when IPP is not obvious. IPP is not anatomically associated with the apex of prostate, and there is no association between IPP and PSM of the apex in the article (p=0.145). Therefore, optimizing the resection strategy at the junction in those without or with slight IPP could possibly reduce the PSM rate of the bladder neck, which of course needs to be further studied.

In the previous studies, the relationship between IPP length and urinary incontinence after RP is relatively clear. In a study involving 119 patients underwent robot-assisted radical prostatectomy, patients with a slight IPP (<5mm) had a higher continence rate than an obvious IPP (>5mm) (38.0% vs 20.8%, p<0.005) in the first month after surgery. However, the significance did not persist from the third month^16^. In another study with more cases (n=821), IPP grade had persistently negative impact on postoperative continence. For example, the incontinence rate in patients without IPP was 8.6%, whereas the rate ranged from 41.7% to 55.6% in different IPP grade groups. On multivariate analysis, IPP was the most powerful predictor of postoperative continence in patients who underwent RALP (p<0.001)^17^.

The study was of limitations. First of all, it is a retrospective study and further subgroup analysis was restricted by the limited case. The findings could be possibly limited by the single source and need to be further verified. Second, we could not assess IPP by MRI because of lacking of electronic MRI images in many patients, given that many patients came to our institution for surgery purpose with printed films from other hospital. Compared with MRI, ultrasound assessment could not provide multidimensional information, such as the shape and the volume of protrusion, which still needs to be further studied. Last but not least, although the present study indicated a more aggressiveness feature in patients with high IPP grade, how IPP influences oncological outcome needs to be further studied.

## Conclusion

IPP is associated with cancer aggressiveness, surgery difficulty and PSM of bladder neck in prostate cancer. Assessment of it provides more information for operations.

## Supplementary Material

Supplementary tables.

## Figures and Tables

**Table 1:** Baseline characteristics and the associations with IPP

Variable	Overall	IPP			P
		No (grade 0)	Slight (grade 1)	Noticeable (grade2)	
Age (years)					<0.001*
Median (IQR)	68 (64-72)	66 (61-70)	67.5 (63-71)	69 (65-73)	
Preoperative total PSA (ng/ml)					0.025*
Median (IQR)	15.0 (8.7-27.0)	12.0 (8.1-23.5)	15.2 (8.0-26.6)	16.0 (9.5-29.3)	
PV, ml					<0.001*
Median (IQR)	30.0 (22.5-44.1)	21.2 (15.8-27.3)	25.5 (20.1-30.8)	40.9 (29.4-54.3)	
Gleason score					**<0.001†**
≤6	111 (14.0)	37 (33.3)	16 (14.4)	58 (52.3)	
7	458 (57.9)	110 (24.0)	115 (25.1)	233 (50.9)	
8-10	222 (28.1)	38 (17.1)	39 (17.6)	145 (65.3)	
pT stage					**0.002†**
≤T2b	203 (25.7)	59 (29.1)	49 (24.1)	95 (46.8)	
T2c	390 (49.3)	92 (23.6)	67 (17.2)	231 (59.2)	
≥T3a	198 (25.0)	34 (17.2)	54 (27.3)	110 (55.6)	
Surgery methods					0.030
Robot-assisted	635 (80.3)	136 (21.4)	141 (22.2)	358 (56.4)	
Open	156 (19.7)	49 (31.4)	29 (18.6)	78 (50.0)	
Involvement of both lobes					**0.009†**
No	233 (29.5)	66 (28.3)	58 (24.9)	109 (46.8)	
Yes	558 (70.5)	119 (21.3)	112 (20.1)	327 (58.6)	
Involvement of micro-nerve fibers					0.077†
No	118 (14.9)	26 (22.0)	17 (14.4)	75 (63.6)	
Yes	673 (85.1)	159 (23.6)	153 (22.7)	361 (53.6)	
Invading prostate capsule					0.81†
No	250 (31.6)	56 (22.4)	52 (20.8)	142 (56.8)	
Yes	541 (68.4)	129 (23.8)	118 (21.8)	294 (54.3)	
Extracapsular extension					0.302†
No	704 (89.0)	168 (23.9)	146 (20.7)	390 (55.4)	
Yes	87 (11.0)	17 (19.5)	24 (27.6)	46 (52.9)	
Involvement of seminal vesicle(s)					**0.035†**
No	605 (76.5)	154 (25.5)	123 (20.3)	328 (54.2)	
Yes	186 (23.5)	31 (16.7)	47 (25.3)	108 (58.1)	
MVI					0.478†
No	748 (94.6)	178 (23.8)	161 (21.5)	409 (54.7)	
Yes	43 (5.4)	7 (16.3)	9 (20.9)	27 (62.8)	
pN stage					0.093‡
N1	29 (3.7)	3 (10.3)	10 (34.5)	16 (55.2)	
N0+x	762 (96.3)	182 (23.9)	160 (21.0)	420 (55.1)	

PV, prostate volume. IPP, intravesical prostatic protrusion. MVI, microvascular invasion. IQR, interquartile range. Variables with p<0.05 are given in bold. * Kruskal-Wallis test. † Pearson's χ2 test. ‡ Fisher's exact test.
