# Supplementary material for: Clinical Significance of Preoperative Assessment of Intravesical Prostatic Protrusion in Radical Prostatectomy
Source: J Cancer. 2023 Sep 11;14(15):2889–94. doi: 10.7150/jca.86582 (PMC10539561; doi:10.7150/jca.86582)
Supplement: Supplementary file 1 — Supplementary tables. [file jcav14p2889s1.pdf]

Table S1. Association between PV and baseline characteristics.

Variable	PV			P
	<30ml	30-50ml	>50ml	
Age (years)				**0.002**
Median (IQR)	68 (63-72)	68 (64-72)	69 (66-73)	
Preoperative total PSA (ng/ml)				**0.004**
Median (IQR)	13.9 (7.7-25.4)	15.0 (9.5-26.5)	17.2 (10.4-34.1)	
Gleason score				**<0.001**
≤6	37 (33.3)	16 (14.4)	58 (52.3)	
7	110 (24.0)	115 (25.1)	233 (50.9)	
8-10	38 (17.1)	39 (17.6)	145 (65.3)	
pT stage				0.224
≤T2b	107 (52.7)	62 (30.5)	34 (16.7)	
T2c	179 (45.9)	136 (34.9)	75 (19.2)	
≥T3a	110 (55.6)	56 (28.3)	32 (16.2)	
Involvement of both lobes				0.424
No	125 (53.6)	70 (30.0)	38 (16.3)	
Yes	271 (53.6)	184 (33.0)	103 (18.5)	
Involvement of micro-nerve fibers				**0.007**
No	53 (44.9)	32 (27.1)	33 (28.0)	
Yes	343 (51.0)	222 (33.0)	108 (16.0)	

Invading prostate capsule				0.672
No	128 (51.2)	75 (30.0)	47 (18.8)	
Yes	268 (49.5)	179 (33.1)	94 (17.4)	
Extracapsular extension				0.939
No	354 (50.3)	225 (32.0)	125 (17.8)	
Yes	42 (48.3)	29 (33.3)	16 (18.4)	
Involvement of seminal vesicle(s)				0.252
No	293 (48.4)	201 (33.2)	111 (18.3)	
Yes	103 (55.4)	53 (28.5)	30 (16.1)	
MVI				0.123
No	381 (50.9)	236 (31.6)	131 (17.5)	
Yes	15 (34.9)	18 (41.9)	10 (23.3)	
pN stage				0.166
N1	12 (41.4)	8 (27.6)	9 (31.0)	
N0+x	384 (50.4)	246 (32.3)	132 (17.3)	

PV, prostate volume. MVI, microvascular invasion. IQR, interquartile range.
Variables with p<0.05 are given in bold.

Table S2. Association of PV with surgery-related indicators and PSM of bladder neck.

	<30ml	30-50ml	>50ml	P
Reconstruction of bladder neck (robot-assisted surgery)	60.8%	60.6%	69.0%	0.251
Surgical bleeding >200ml	33.3%	38.9%	27.8%	0.310
Blood transfusion	0.8%	0.8%	1.4%	0.782
Postoperative hospital stays >14d	6.6%	8.3%	9.9%	0.403
PSM of bladder neck	11.1%	13.4%	9.9%	0.532
PV, prostate volume. PSM, positive surgical margin.				
